# Human Satellite 1A analysis provides evidence of pericentromeric transcription

**DOI:** 10.1186/s12915-023-01521-5

**Published:** 2023-02-08

**Authors:** Mariana Lopes, Sandra Louzada, Daniela Ferreira, Gabriela Veríssimo, Daniel Eleutério, Margarida Gama-Carvalho, Raquel Chaves

**Affiliations:** 1 CytoGenomics Lab, Department of Genetics and Biotechnology (DGB), University of Trás-Os-Montes and Alto Douro (UTAD), 5000-801 Vila Real, Portugal; 2 BioISI – Biosystems & Integrative Sciences Institute, Faculty of Sciences, University of Lisboa, 1749-016 Lisbon, Portugal

**Keywords:** HSat1A, Pericentromere, Satellite transcription, Transcript polyadenylation, Noncoding RNA

## Abstract

**Background:**

Pericentromeric regions of human chromosomes are composed of tandem-repeated and highly organized sequences named satellite DNAs. Human classical satellite DNAs are classified into three families named HSat1, HSat2, and HSat3, which have historically posed a challenge for the assembly of the human reference genome where they are misrepresented due to their repetitive nature. Although being known for a long time as the most AT-rich fraction of the human genome, classical satellite HSat1A has been disregarded in genomic and transcriptional studies, falling behind other human satellites in terms of functional knowledge. Here, we aim to characterize and provide an understanding on the biological relevance of HSat1A.

**Results:**

The path followed herein trails with HSat1A isolation and cloning, followed by in silico analysis. Monomer copy number and expression data was obtained in a wide variety of human cell lines, with greatly varying profiles in tumoral/non-tumoral samples. HSat1A was mapped in human chromosomes and applied in in situ transcriptional assays. Additionally, it was possible to observe the nuclear organization of HSat1A transcripts and further characterize them by 3′ RACE-Seq. Size-varying polyadenylated HSat1A transcripts were detected, which possibly accounts for the intricate regulation of alternative polyadenylation.

**Conclusion:**

As far as we know, this work pioneers HSat1A transcription studies. With the emergence of new human genome assemblies, acrocentric pericentromeres are becoming relevant characters in disease and other biological contexts. HSat1A sequences and associated noncoding RNAs will most certainly prove significant in the future of HSat research.

**Supplementary Information:**

The online version contains supplementary material available at 10.1186/s12915-023-01521-5.

## Background

Satellite DNA (satDNA) sequences consistently organize in arrays of tandem repeats, preferentially located at (peri)centromeric and subtelomeric heterochromatin [1, 2]. Historically, these sequences were acknowledged by distinguishable satellite bands in cesium chloride gradients of human genomic DNA [3] and termed classical satellites I, II, and III [4–6], today known as human satellite families HSat1, HSat2, and HSat3. The human reference genome (current patch GRCh38.p14) is still an undeniable hostage of assembly issues related with acrocentric HOR/pericentromeric sequence sharing, evidently under- or misrepresenting satDNA sequences [7]. From a closer look into Dfam [8] or Repbase [9], HSat1 can be found in two different annotation types: SAR (DF0001062.4), firstly acknowledged to repeat in 42-bp units, composed of two alternating repeat unit types, A (17 bp) and B (25 bp) [6], and described with a probe (pTRI-6) locating at chromosomes 3 and 4 and acrocentric chromosomes (chr13, 14, 15, 21, and 22) [10, 11]; and HSATI (DF0000210.4), formerly identified as male-specific and containing one Alu family member [12]. Addressing the high number of gaps in the human reference assembly related with acrocentric p-arms, the Telomere-to-Telomere (T2T) consortium has recently released the T2T-CHM13 human genome assembly [13], of which HSat1 constitutes 0.47% of the total sequence [14]. In this work, HSat1 was re-classified into HSat1A and HSat1B elements. HSat1A (corresponding to SAR), the main scope of the present paper, was predominantly found to form longer tandem repeats of the 42-bp monomers: 378, 751, 3013, 3474, and 6330 bp [14, 15].

Despite being a part of constitutive heterochromatin, human satellites (HSats) are not transcriptionally inactive [16]. HSat transcription into satellite noncoding RNAs (satncRNAs) is reported as bidirectional and promoted by RNA polymerase II (Pol II) [17]. Pericentromeric satellite transcription was reported as strand-specific depending on cell state (e.g., stress versus senescence) [18, 19]. As products of Pol II transcription, ncRNAs regulation is dependent of cotranscriptionally occurring RNA processing. Polyadenylation consists in the 3′ processing of mRNAs or ncRNAs through the addition of poly(A) tails, known to influence RNA stability and transport [20, 21]. Shortly, when poly(A) signals emerge during nascent transcription, 3′-end cleavage and polyadenylation (CPA) complex is recruited, inducing Pol II slowdown and transcription termination. Transcripts of variable lengths can therefore result from alternative polyadenylation (APA) using different poly(A) sites (PASs) [22]. Premature CPA (PCPA) can result from the selection of proximal PASs, especially in highly proliferative cells [23]. PCPA is strictly balanced by its suppressing counterpart—a process termed telescripting, which essentially assures full transcript length. Therefore, both mRNA and ncRNA transcript variability depends on the regulation of the PCPA-telescripting duality [24].

Many ncRNAs are transcriptionally dysregulated in disease states: in transcript levels [25] and potentially in transcript processing, or both. Increased satDNA expression has been related with genomic instability [26]. While preserving chromosome integrity [27–29], tandem repeats can potentially represent beacons of instability in cancer genomes [30]. Chromosome breaks tend to occur in pericentromeric satellite regions [31], possibly altering the regulation of transcription of satellite sequences [32, 33]. By its turn, available pericentromeric chromatin in cancer might also predispose chromosomes to break [34]. Hence, cancer progression can be related with the emergence of chromosomal alterations, caused by (or causing) changes in genomic architecture and transcription deregulation in noncoding regions [34, 35].

ncRNAs, and more precisely satncRNAs, expression is underrepresented in transcriptomic data [36], as ncRNAs seem to pose methodological and analytic challenges, relating with their complex diversity and repetitive nature [37]. Thus, the function of the human noncoding genome and satellite transcription has been gradually addressed. Centromeric αSat (alpha satellite) transcripts in particular have been considered vital for kinetochore stabilization and centromere cohesion [38–41]. The progressive description of pericentromeric HSat2 and HSat3 transcripts has elevated their status to essential in several cellular contexts [42], like the formation and regulation of heterochromatin [43, 44], aging [18, 45], response to stress [19, 46, 47], differentiation [48, 49], and cancer [26, 50, 51].

Comprehensive work describing pericentromeric HSat2 and HSat3 has been presented over recent years. However, HSat1A lacks molecular and cytogenomic studies. To characterize HSat1A, we present copy number variation and transcriptional analysis in distinct cell lines (tumoral and non-tumoral), along with single-cell analysis by RNA-fluorescence in situ hybridization (RNA-FISH). This study is coupled with cytogenetic mapping and immunofluorescence, as well as in silico HSat1A assessment. We also performed 3′ RACE-seq and pointed some hints to the inclusion of HSat1A into the ncRNA landscape. To the best of our knowledge, this work constitutes, so far, the deepest analysis of pericentromeric HSat1A, the most AT-rich fraction of the human genome.

## Results

### HSat1A isolation, copy number analysis, and expression profiling

**Fig. 1:**
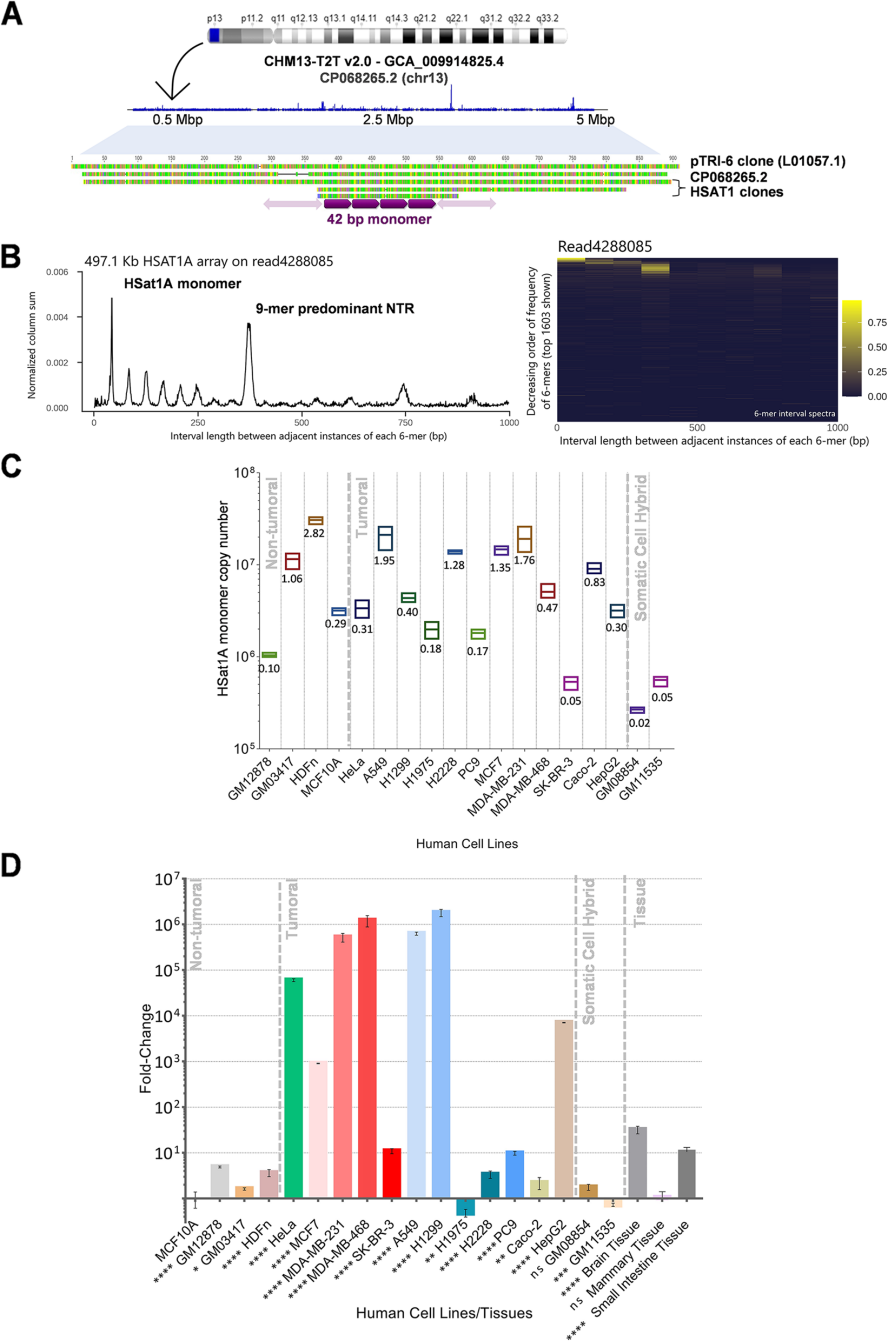
HSat1A sequence analysis and copy number/expression evaluation. **A** Obtained HSat1A clones (GenBank accession numbers: OP172545–OP172627) were analyzed in Tandem Repeats Finder and proved to be systematically composed of 42-bp repeats. HSat1A clone was BLAST searched against CHM13-T2T v2.0 (GenBank assembly accession GCA_009914755.4) and filtrated hits were mapped into chromosomes. HSat1A BLAST hits are represented (in blue) in CHM13-T2T chromosome 13 (CP068265.2), reported to have a large HSat1A array [14]. The ideogram was adapted from the Ensembl genome browser. In silico mapping of HSat1A hits was performed in Geneious. Concatenated hits are observable in a 5-Mb extent. HSat1A clones, HSA13 T2T HSat1A array [13, 64], and pTRI-6 (L01057.1) sequence stretches were aligned (Additional file 1: Supplementary Table S3). **B** HSat1A periodicity spectrum and heatmap in GM12878 sequencing data. NTRprism reveals two predominant peaks: one corresponding to HSat1A monomer and the second to a 9-mer higher repeat. **C** HSat1A monomer copy number quantification in several human cell lines. Values are mean ± SD (*n* = 3) (Additional file 4). Statistical analysis is detailed in Additional file 1: Supplementary Fig. S2. HSat1A estimation in percentage of the haploid human reference genome (bp/total bp). **D** HSat1A ncRNA relative quantification by RT-qPCR in fold change (MCF10A set as reference). Values are mean ± SD (*n* = 3) (Additional file 4). *p ≤ 0.05, **p ≤ 0.01, ***p ≤ 0.001, ****p ≤ 0.0001, ns, not statistically significant (one-way ANOVA with Tukey’s multiple comparisons test)

The characterization of HSat1A is hampered by the low amount of information related with this satellite and its sequence, stemming in part from technical constrains associated to its AT richness and low read coverage in sequencing studies. Therefore, we decided to take a more classic approach and begin by performing HSat1A PCR isolation followed by cloning and Sanger sequencing (Additional file 1: Supplementary Table S1), having obtained clones with a mean of 87.9% identity, composed of 42-bp repeats, and a high AT content (77% on average) (Fig. 1A), as expected for HSat1A. Choosing one of the smaller clones as representative of the family (HSat1A clone from herein), we next checked HSat1A chromosomal locations: in GRCh38.p14, HSat1A sequences mapped to chromosomes 3, 4, 8, 14, and 22; in CHM13-T2T v2.0, matching positions were present in chromosomes 3, 4, 8, and all acrocentric chromosomes (Additional file 2). When broadly querying nucleotide databases, most of the obtained hits belong to unlocalized sequences (Additional file 1: Supplementary Fig. S1, Additional file 3), which points to the recurrency of unplaced tandem repeats (like HSat1A) in attempts to assembly the human genome.

As satDNA genomic occurrence and persistence can be determined by long-range organization and structure [52], we further addressed HSat1A periodicity from publicly available data. As mentioned, the analysis of the CHM13-T2T assembly with the NTRprism software tool, specifically developed for that purpose, reported that HSat1A presents a higher-order organization with the predominance of 9-mer NTRs (nested tandem repeats) [14]. To expand on this information and assess the recurrency of HSat1A organization in a reference/non-tumoral cell line used throughout this paper, we applied the same approach to nanopore long-read sequencing data from GM12878 cell line (Utah/CEPH pedigree) [53]. The most frequent periodicity identified corresponded to the HSat1A 42-bp monomer, followed by the 378-bp 9-mer array (Fig. 1B).

Generally, tandem repeats are underrepresented in databases [54, 55], leading to a gross undervaluation of their genome representativeness. The evaluation of copy number fluctuation between different genomes is particularly interesting as it can provide insights into the evolutive behavior of a given tandem sequence. Given the lack of information and wide-ranging molecular studies on HSat1A, we next evaluated the copy number of this sequence in sixteen human cell lines (tumoral and non-tumoral; Additional file 4) from different tissues, by qPCR. Additionally, we assessed the HSat1A copy number in two human/murid somatic cell hybrids for human chromosomes 14 and 21. Monomer copy number showed considerable variability between cell lines, which seemed to be independent of tissue type and origin (tumor-derived or non-tumoral) (Fig. 1C, Additional file 4). For example, MDA-MB-231, MDA-MB-468, or MCF7 (cell lines derived from breast adenocarcinomas) all have a significantly higher copy number than MCF10A (non-tumorigenic), but the latter presents more copies than SK-BR-3 (breast adenocarcinoma). The same pattern of variation can be observed in the lung cancer cell line repertoire assessed in this study (H1299, A549, H1975, H2228, and PC9). Given the well-known connections between genome instability and human satDNAs [56], it would be reasonable to expect HSat1A CNV to differ between normal and tumor cell lines. Nonetheless, the polymorphic nature of these sequences (greatly varying even between individual arrays) and their natural behavior as sources of CNV in genome evolution [1, 31, 57, 58] limit any assessment of HSat1A CNV contribution to instability in the absence of information about the starting point. In fact, satellite CNV is often so substantial that it is cytogenetically detectable between individuals [59]. In addition to the observed high HSat1A copy number variation (CNV) in non-tumoral cell lines (MCF10A, GM12878, HDFn, and GM03417), the analysis of somatic cell hybrids (GM11535 and GM08854) also seems to underscore the relevance of populational polymorphism. Indeed, from early studies, HSat1A was reported to be largely present in chromosomes 13 and 21 and less represented in other acrocentric chromosomes [10]. However, we find that the GM11535 cell line, with a single copy of human chromosome 14 per cell, presents a higher HSat1A copy number than GM08854, which has 1–5 copies of human chromosome 21/cell. The discrepancy between these results and past observations could be explained by significant inter-individual variation in the size of HSat1A arrays. Excluding somatic cell hybrids, HSat1A genome representativity in the analyzed human cell lines ranges between ~ 0.05 and 2.82%, with an average of 0.83% (Fig. 1C). These results are in line with the estimation of 0.43% for HSat1A sequences in the CHM13-T2T genome assembly. To further assess the reliability of our qPCR quantification, we compare our results for the GM12878 cell line with the available sequencing data using RepeatMasker [60]. The estimated genomic abundance based on the sequencing dataset was of ~ 0.2% (Additional file 1: Supplementary Table S2), within close range of our qPCR estimate of ~ 0.1% for the same cell line. Thus, it is fair to assume that the qPCR quantification method used in this work is able to estimate HSat1A representativity with an accuracy comparable to the estimates obtained from sequencing data analysis.

Many lncRNAs, and more specifically satncRNAs, have been linked to tumor formation and progression, essentially by their abnormal level of transcription [61, 62]. Thus, we next evaluated HSat1A transcription in the same set of cell lines used for copy number assessment by RT-qPCR. Additionally, we quantified HSat1A transcripts in three human tissues (mammary, small intestine, and brain). The collection of cell lines derived from breast adenocarcinomas (MCF7, MDA-MB-231, MDA-MB-468, and SK-BR-3) and the one derived from non-small cell lung cancer (H1299, A549, PC9, H1975, and H2228) presented variable transcriptional profiles (Fig. 1D). When comparing to MCF10A, some cancer cell lines (MDA-MB-231, MDA-MB-468, H1299, and A549) have aberrant overexpression of HSat1A, while others (SK-BR-3, PC9, and H2228) still overexpress HSat1A, but to a much smaller extent. This comparison is particularly relevant because of the same tumor type of the analyzed cell lines. Such varying levels of transcription in similar cancer tissues question the premise of an analogous overexpression behavior for HSat1A ncRNA [26, 63] or at least presuppose a more complex regulatory scenario. Other cancer cell lines—HeLa and HepG2—show transcription upregulation, while Caco-2 has lower HSat1A transcription. Non-tumoral cell lines—GM12878, GM03417, or HDFn—have more HSat1A transcripts than MCF10A. Nevertheless, none of the analyzed non-tumoral cell lines shows a transcription level comparable with the aberrantly expressing cancer cell lines (e.g., MDA-MB-468 or HeLa). By analyzing HSat1A ncRNA in human tissue samples, we could observe a non-statistically significant difference between MCF10A (originated from mammary gland) and mammary tissue (as expectable), but a higher transcript level in small intestine and brain tissues.

The comparison between copy number and expression in the analyzed cell lines results in no significant correlation between the two (Spearman’s correlation with *r* = 0.4, *p*-value (two-tailed) = 0.09).

#### HSAT1 physical mapping

**Fig. 2:**
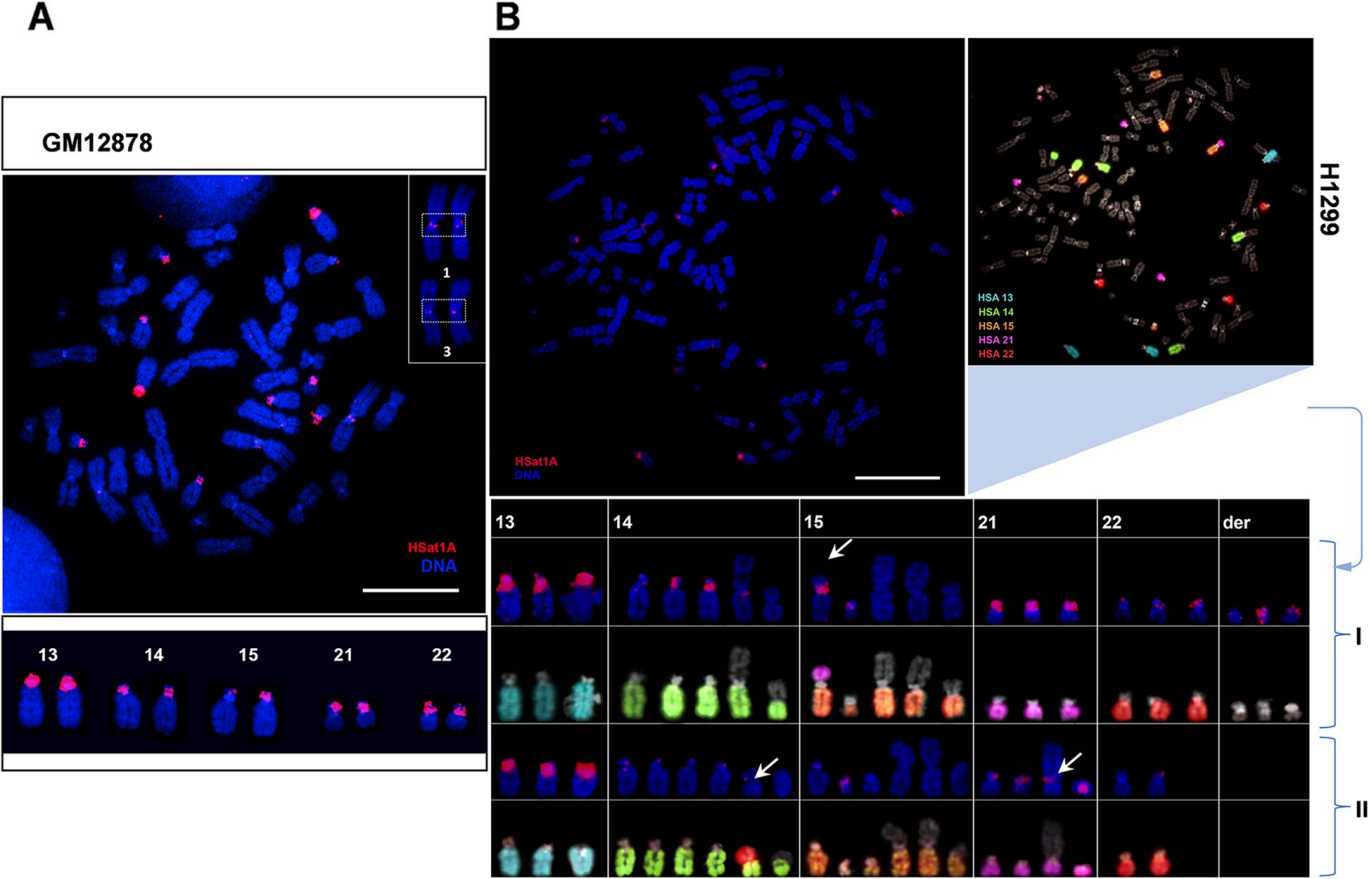
HSat1A FISH mapping (red) in human chromosomes (blue). **A** HSat1A mapped in GM12878. Obtained hybridization signals in acrocentric chromosomes are highlighted above. Hybridization signals are also present in chromosomes 1 and 3. Chromosomes were identified by reverse-DAPI. **B** HSat1A mapped in chromosomes from H1299, sequentially hybridized with human painting probes for acrocentric chromosomes. Corresponding chromosomes are visible in the table above (two different clones). The illustrative metaphase corresponds to clone I (first two rows). The column “der” for clone I shows three derivative chromosomes (non-acrocentric) with visible HSat1A signals. White arrows indicate chromosomal alterations occurring with acrocentric chromosomes and modifying the HSat1A hybridization pattern. Scale bars represent 10 μm

We next proceeded to physically map isolated HSat1A clones in human chromosomes and compare the corresponding locations with available data. In Fig. 2A, we present FISH (fluorescent in situ hybridization) results showing HSat1A mapping in a metaphase preparation from GM12878. The tested subset of HSat1A clones (representative of the three obtained sizes) showed very similar hybridization patterns in the same pericentromeric sites. Likewise, HSat1A chromosomal locations were similar between cell lines (not shown). Clear hybridization signals (with different intensities) were observed in all acrocentric chromosomes. Moreover, we detected FISH signals in chromosomes 1 and 3. The former location (chr 1) is reported here for the first time (as far as we know) (Additional file 1: Supplementary Fig. S3).

We further mapped HSat1A in the tumor cell line H1299, in which we detected the highest level of HSat1A ncRNA transcripts. After HSat1A probe hybridization, we performed sequential FISH with painting probes for human acrocentric chromosomes. Figure 2B illustrates two different clones found in this cell line, both with a clear presence of chromosomal alterations (e.g., translocations), evidently affecting the intensity/presence of HSat1A hybridization signals. Given these results, CNV between H1299 clones is also likely to be happening. The cytogenetic mapping analysis points to a possible role of HSat1A as a player in genomic/chromosomal instability and a plausible fragile pericentromeric location, warranting further exploration.

#### HSat1A transcripts: cellular patterns

**Fig. 3:**
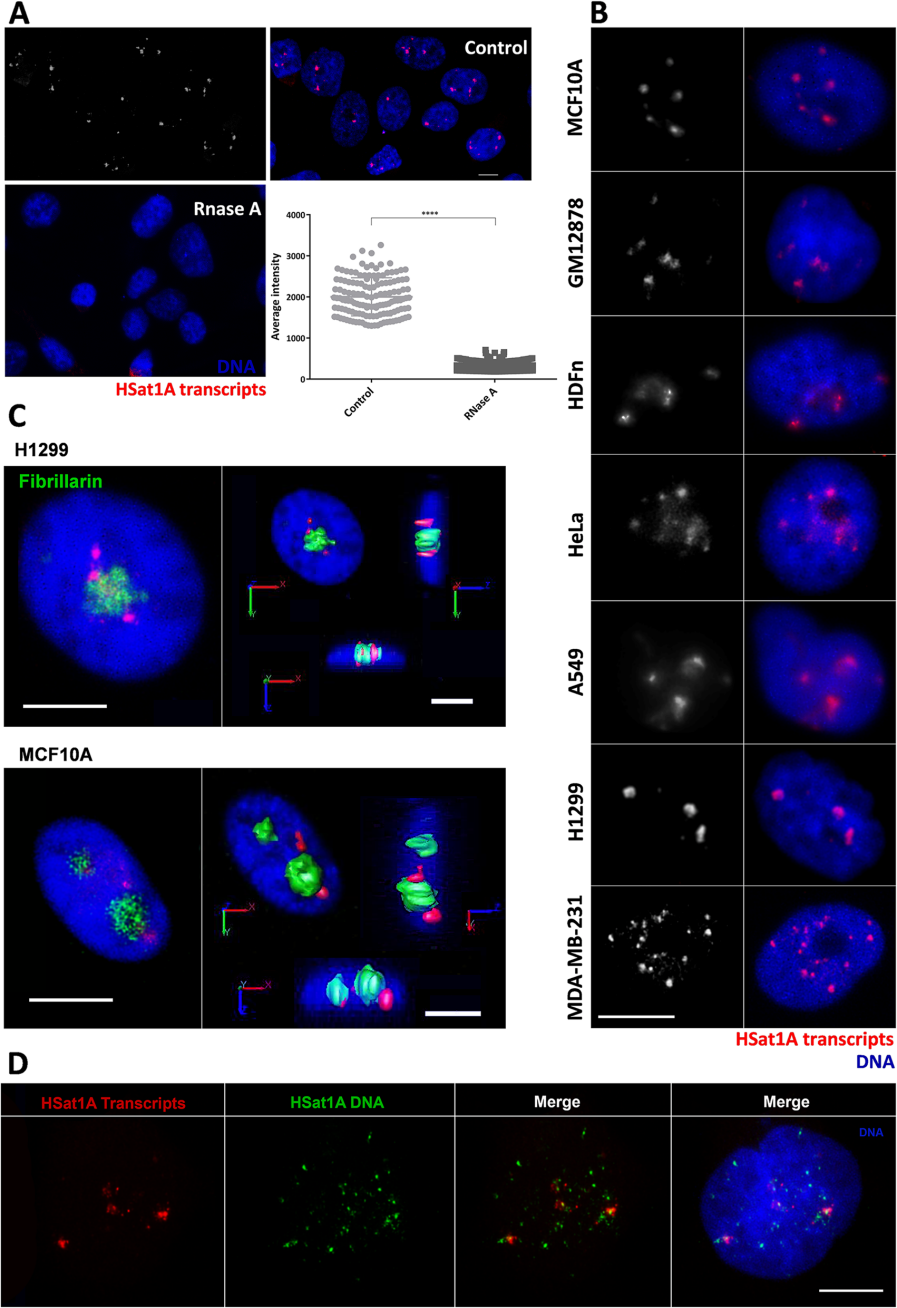
Detection of HSat1A transcripts by RNA-FISH and RNA-FISH/IF. **A** HSat1A RNA-FISH with RNase A treatment. HSat1A transcripts were detected by RNA-FISH (red) in control and treated cells. Signal decrease in RNase-treated cells demonstrates that the observed signals are RNA-specific. Evaluation of the average intensity of active signal objects (all slices) in RNA-FISH control and RNA-FISH + RNase A was performed in “Counting and Tracking” (AutoQuant X3). Analysis shown in H1299. Values are mean ± SD (*n* = 20). ****p ≤ 0.0001 (unpaired *t* test). **B** Nuclear organization of HSat1A transcripts (red). Different spatial distribution and number of foci are observable between cell lines with similar amounts of HSat1A transcripts (RT-qPCR data). **C** Spatial organization of HSat1A transcripts in relation to nucleoli. HSat1A RNA-FISH (red) coupled with IF for fibrillarin detection (green). HSat1A transcripts seem to accumulate adjacently to nucleoli, as seen by confocal 3D image analysis for H1299 and MCF10A cells. Orthogonal slices for axis projections are displayed with isosurfaces for both channels. **D** HSat1A RNA-FISH (red) followed by HSat1A DNA-FISH (green). Merged confocal images show distinct signal features, with some co-localized signals. DNA is in blue (DAPI) in all the presented images. Scale bars represent 10 μm

To further characterize HSat1A transcription, we proceeded to determine the subcellular localization of HSat1A transcripts by RNA-FISH, an approach that has been used to visualize and analyze the spatial distribution of ncRNAs in several species and conditions [17, 46, 63–65]. After detecting HSat1A ncRNA foci, we performed RNase A treatment prior to hybridization, to ensure signal specificity and exclude unintentional DNA hybridization (analysis shown in H1299 cells). RNA-FISH signals decreased significantly, as also seen by the evaluation of the average intensity of active fluorescent objects in control RNA hybridization and RNase treatment (Fig. 3A; Additional file 1: Supplementary Table S4). Detection of HSat1A transcripts in different cell lines allowed to perform a single-cell topographic observation of the obtained signals (Fig. 3B). RNA-FISH is presented in seven cell lines to show feasibly distinctive transcript features between cell lines with similar amounts of HSat1A transcription. Standard streptavidin-Cy3 detection was used in highly expressing cell lines. Cell lines with reduced HSat1A transcription required signal amplification (tyramide signal amplification). HSat1A transcripts exhibit cluster-like organization and consistent nuclear localization, though with distinct signal patterns in different cell lines. For example, HeLa, H1299, and MDA-MB-231 cells (all aberrantly expressing HSat1A) have evident differences concerning the number and spatial distribution of HSat1A ncRNA foci. Moving towards in our approach, we studied the nuclear localization of HSat1A transcripts, coupling HSat1A RNA-FISH with immunofluorescence (IF) for the detection of fibrillarin. Our intent was to compare nucleoli localization with HSat1A ncRNA signal clusters. Image 3C illustrates this analysis in H1299 and MCF10A, where HSat1A transcripts can be spotted contiguously to nucleoli (peripheric). Confocal 3D images allow to visualize signal spatial distribution (with isosurfaces and orthogonal slices for axis projections). We also performed sequential DNA-FISH to the slide with RNA-FISH, imaged the same slide fields, and merged both confocal images (Fig. 3D). HSat1A signals are differently organized in both FISH experiments, even though some RNA signals colocalize with HSat1A DNA, pointing to nascent HSat1A transcription.

#### Scanning the features of HSat1A transcripts

Our approach to analyze HSat1A transcripts followed by searching HSat1A clones in NCBI SRA data, specifically data from PRJNA362590 (HeLa PacBio ncRNA-Seq; SRA experiment: SRX2505545) [66, 67]. We obtained 307 hits for 128 target sequences, with a percent identity superior to 80.8% and multiple reads bigger than 1 kb. For verifying the possible existence of annotated lncRNAs with similarity with HSat1A, we next BLAST searched HSat1A against LNCipedia Version 5.2 Full Database [68] (Additional file 5). Our results retrieved three NONCODE v4 annotated transcripts with two exons: lnc-RNF170-2:1 (475 nt, sense intronic ncRNA), lnc-RNF170-1:1 (223 nt; lincRNA; NONCODE Gene ID: NONHSAG050120.2), and lnc-RNF170-5:1 (629 nt; lincRNA; NONCODE Gene ID: NONHSAG050119.2). By analyzing UCSC Genome Browser [69] on human (GRCh38/hg38), annotated ncRNAs all map to chromosome 8, in overlapping locations with annotated SAR (RepeatMasker [60]) and three ESTs (Expressed Sequence Tags) from GENCODE v39 [70]. The latter sequences were found in nervous, liver, and testis tissue [36]. The percentage of identities with HSat1A clone were ~ 80%.

**Fig. 4:**
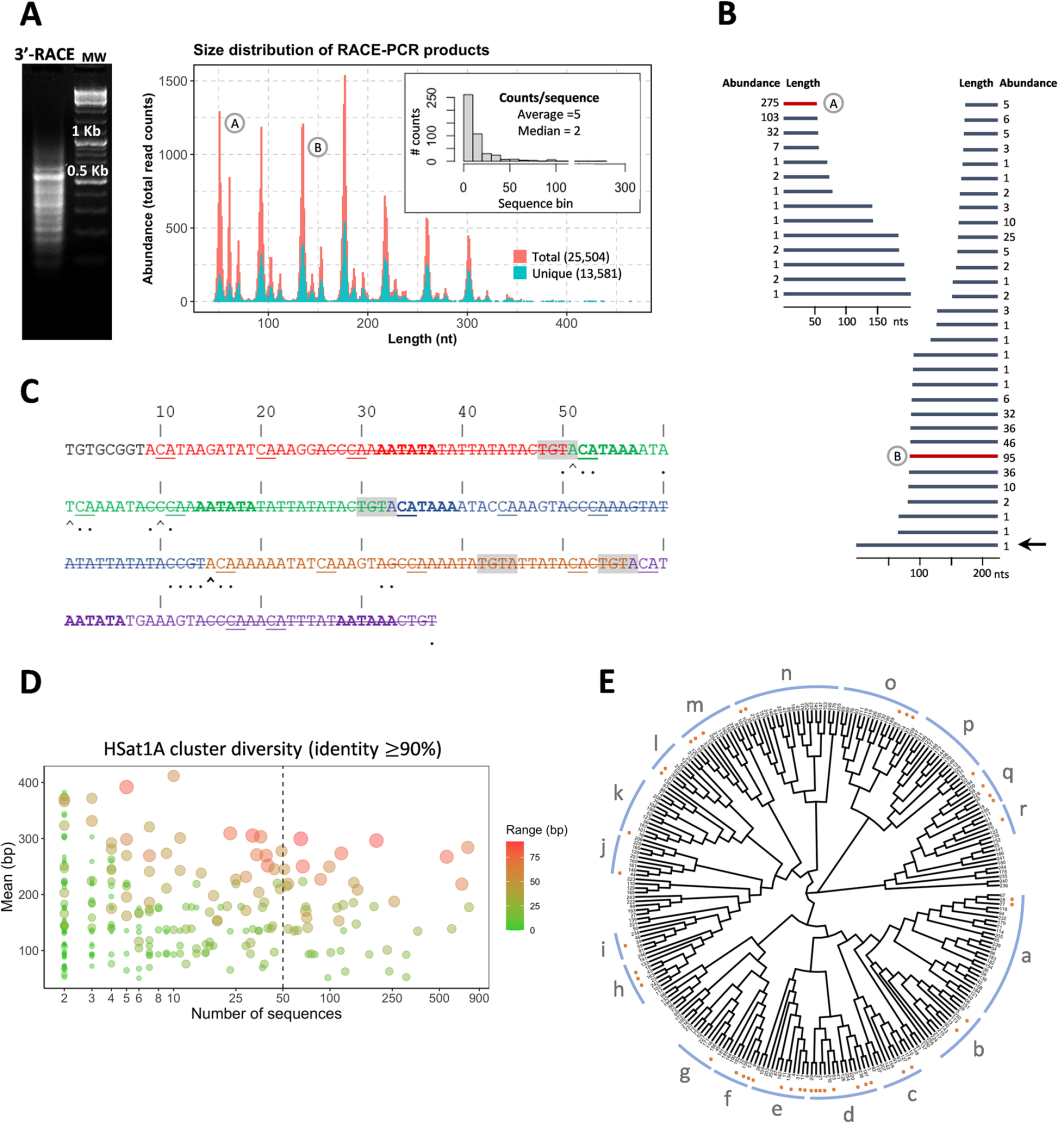
HSat1A 3′ RACE analysis. **A** Agarose gel corresponding to HSat1A 3′ RACE-amplified transcripts (left); molecular weight (right). A size distribution plot is presented for the graphical representation of HSat1A reads. Assembled transcripts contained HSat1A peaks corresponding to multiples of the 42-monomer. From the total of HSat1A sequences, 16,332 sequences were found to be unique (blue in plot). The bar chart (top right corner) shows the high representation of unique sequences, visible in the distribution of counts/sequence. A and B (round) sequences are representative of the identified peaks and are displayed in B. **B** HSat1A tandem transcript organization. In a universe of 200 nucleotides, it is possible to reconstruct transcripts of longer lengths with smaller sequences. The black arrow points to the longer represented read (structure explored in **C**). **C** HSat1A read structure analyzed in the light of the consensus mammalian poly(A) signal. Different colors display HSat1A monomers HSat1A are organized in alternative A (17 nt) and B (25 nt); strikethrough nucleotides in the figure. Sequences that may function as poly(A) signal hexamers [72] are highlighted in bold. Shades of gray correspond to the sequence that functions as the recognition of the poly(A) signal in the absence of the canonical hexamer element [A(A/U)UAAA]. Nucleotides located at the site of optimal 3′ cleavage, named the poly(A) site, are underlined. Arrows point to the largest number of duplicates that are cleaved at that nucleotide position (bold for the largest most abundant). Dots represent the cleavage location of duplicates that contain a difference ≥ 1 nucleotide from the previous sequence. The cleavage positions address the possible occurrence of alternative polyadenylation, resulting in the observable variation of transcript length. **D** HSat1A transcript cluster membership. Colors determine the range (bp) between sequences of the same cluster. **E** Phylogenetic tree depicting transcript variability, constructed from the multiple alignment between the center sequences of each cluster. Clusters can be grouped accordingly to their distance (groups a–r). Orange dots represent clusters with more than 50 elements

In order to have a deeper understanding of HSat1A transcripts, we performed 3′ RACE (rapid amplification of cDNA ends) [71] in HeLa RNA using an oligo-dT anchor primer for reverse-transcription and a forward PCR primer targeting the HSat1A sequence. Analysis of 3′ RACE products by agarose gel electrophoresis revealed a ladder of products, with the most intense band around 550 bp (Fig. 4A; Additional file 1: Supplementary Fig. S4). This result suggests that HSat1A transcripts are polyadenylated and thus likely transcribed by RNA polymerase II [72]. To characterize the amplified products, we performed a 300-nt paired-end high-throughput sequencing using the Illumina MiSeq platform. A total of ~ 1 × 10^5^ reads (Additional file 1: Supplementary Table S5) were obtained (for R1 and R2), which were quality and size filtered and assembled into ~ 3.5 × 10^4^ complete 3′ RACE transcripts, as described in the “Materials and methods” section. Approximately 70% of these assembled transcripts presented the HSat1A motif and were distributed across a size range of 51 to ~ 400 nucleotides, with peaks corresponding to multiples of the 42-monomer size (Fig. 4A). Although we cannot exclude PCR size-amplification bias, the most prevalent sequence size was ~ 170 nt. Within this universe, 16,332 sequences were found to be unique, attesting to the high complexity of the HSat1A transcriptome. By analyzing sequence reads in a window of 200 nucleotides, we could observe a progressive reconstruction of longer reads by piecing together smaller ones (Fig. 4B, Additional file 6), possibly suggesting mechanisms of ACPA. To test this theory, we examined the structure of a representative read, having found multiple alternative and non-canonical PASs [73], organized in a known poly(A) signal structure [74], and cleavage sites often corresponding to the actual read lengths. To get a better perspective of the degree of sequence variability, we clustered this dataset into groups with a minimum sequence identity of 90%, identifying a total of 257 clusters, 50 of which had more than 50 elements (Fig. 4D, E). We next unbiasedly searched for sequence motifs within each of the 50 mentioned clusters. We found that the obtained repeated motifs invariably compose, or were composed of, HSat1A 42 nt monomers (Additional file 1: Supplementary Fig. S5; Additional file 6).

## Discussion

From old parallelisms with “darker,” “unknown,” or “useless” contexts, satDNA has been growing amongst research interests, especially when related analysis obstacles are progressively being outpaced. Centromeric and pericentromeric satellite sequences were effectively difficult to incorporate in genomic assemblies, as linearly order long arrays of tandem repeats was technically unachievable [75–77]. This situation is observable in the initial part of this paper: isolating and subsequently mapping HSat1A clones in the human reference genome GRCh38.p14 highlighted the still significant assembly gaps in reference acrocentric/pericentromeric regions. When comparing reference mapping with our FISH results in GM12878 (Fig. 2A), we can clearly see that HSat1A should be highly represented in in silico maps of chromosomes 13 and 21 (intense FISH signals but absent in reference sequences). Indeed, the entire p-arms of acrocentric chromosomes (and therefore, HSat1A repeats) represented long stretches of gaps, until the emergence of CHM13-T2T [78]. The difficulties in assembling HSat1A repeats (AT-rich and highly shared between acrocentric chromosomes) were experienced while sequencing acrocentric p-arms [13] and are observable in our BLAST search returning 61.2% of unlocalized sequences. CHM13-T2T was an unquestionable addition to satDNA studies—our HSat1A clone mapping in CHM13-T2T (Additional file 2) was consonant with our FISH mapping in acrocentric chromosomes (Fig. 2). FISH signals were also observable in chromosomes 1 and 3. In particular, HSat1A mapping in chromosome 1 (not previously reported) points to the need of inquiring human satDNA variation. Also, it suggests the pertinence of using cytogenetic mapping in both clinical and non-clinical contexts.

SatDNA sequences can represent up to 10% of the human genome [33, 79] and have a major representation of CNVs, even between individuals [1, 31, 57]. These changes in satellite copy number may occur through arrays contractions/expansions, due to recombination between repeats with unequal change [29, 57]. HSat1A monomer copy number quantification in a set of human cell lines likely features this polymorphism as a source of human variation. For example, αSAT representativity can vary between 1 and 5% and HSat2/3 between 1 and 7% (different human populations) [59, 80]. So, the observed differences in HSat1A copy number between cell lines (of 2.5% in some cases) were quite expectable and might not be related with the characteristics of each cell line and/or the use of cancer cell lines. Our monomer copy number analysis presents a limitation: our genome percentage estimation is based on the size of the haploid human reference genome and does not refer to the specific karyotype of each cell line, certainly harboring structural rearrangements and/or aneuploidies. We chose this method of quantification to directly compare cell lines, since all of them are normalized to the haploid human reference genome. Despite still providing new and accurate (comparable with sequencing estimation) information about HSat1A CNV, our work would benefit from coupling with other quantitative methods like digital droplet PCR. Satellite CNV in cancer can be particularly advantageous for polymorphic variation between cell populations and rapid evolution [30]. However, human genome variation might be in play here, which calls for the need of assessing satDNA CNV in a pangenomic approach [81, 82], in order to evaluate its functional significance in diverse states, evidently assuming that we might still be underestimating the genomic representation of these sequences. Besides the variation found between individuals, HSat1A arrays might still vary in size between homologous chromosomes, since heterochromatic regions possess a high level of polymorphism [82, 83]—visible in HSat1A signals in Fig. 2. Hence, this kind of heteromorphism can also contribute to a limited understanding of HSat1A copy number in the context of chromosome representativity and haplotype variation.

By turn, (peri)centromeric transcription has been related with cancer [17, 26, 51, 62, 83]. HSat1A transcription was assessed in the same cell lines used for copy number analysis, yet with even more dissimilar results. So, in concordance with the possible polymorphic nature of this sequence, there was no clear evidence of an association between HSat1A copy number and expression, as already seen in other works with satellite sequences [84]. When quantifying HSat1A transcripts, we analyzed cancer cell lines from similar origins with astounding different profiles. It should be noted that our RT-qPCR quantification is of relative nature, probably masking transcription features related with transcript size variability and cell heterogeneity. However, by comparing between cell lines, our analysis hints for different transcription patterns, despite not allowing a precise transcript quantification. Later on, applying single-cell sequencing technologies would certainly favor satncRNA research.

In cancer, ncRNAs and tumor suppressor genes/oncogenes share the trait of highly variable transcription, which can indicate the presence of post-transcriptional complexed-regulated pathways [85]. Furthermore, the tendency for mutation of genomic regulators in diseases such as cancer could have a determining role in ncRNA transcription deregulation [85–87]. Thus, the variable genetic and epigenetic landscape of HSat1A might be determining transcription differences. For example, changes in histone methylation, particularly in the levels of H3K9me3 (a typical repressive mark found in satellite heterochromatic regions) [88, 89] can relate with cancer predisposition [90, 91]. If HSat1A transcription turns to be a player in genome instability and epigenetic regulation, more functional studies are needed in order to place the transcriptional event—causing changes in epigenetic regulators [50] or being deregulated as a consequence. This duality can also be related with cancer chromosomal instability, as shown in Fig. 2B. Chromosome breaks are more prone to occur in satellite pericentromeric regions [31], causing chromosomal rearrangements with the ability to compromise genome stability [56] and altering the transcription status of genes or even satellite sequences themselves [32, 33, 92].

Through RNA-FISH experiments in multiple cell lines, we subsequently address the subcellular localization of these transcripts and found that they are nucleus-specific, despite having different signal topographies in different cell lines. It became clear that HSat1A transcription deregulation does not result in altering transcript location, although possibly resulting in a different organization within the nucleus. HSat1A RNA-FISH together with fibrillarin staining allowed to address a perinucleolar presence to HSat1A ncRNA foci. The signal distribution of HSat1A can be linked with the organization of satellite DNA and RNA into chromocenters, often associating in close proximity to the nucleoli and/or nuclear membrane [93, 94]. Still, epigenetic alterations and/or changes in nuclear architecture could explain different patterns, like the one seen in HSat1A transcripts from MDA-MB-231 (Fig. 3C).

3′ RACE allowed us to perform a deeper characterization of HSat1A transcripts by proving the accumulation of polyadenylated ncRNAs. Other reports show that polyadenylated pericentromeric transcripts can be detected in humans [48]. Polyadenylation of Pol II ncRNA transcripts can be tightly related with transcription termination and intensively regulated in the presence of cellular stresses and/or cancer-associated mutations [22, 95]. The size variability of HSat1A transcripts, also attested by the presence of multiple PASs, is likely to be a result of APA. APA can be possibly regulated by the levels of U1 telescripting (when high, inhibiting PCPA; when low, resulting in smaller transcripts) [96]. Moreover, poly(A) site selection becomes more complex in the presence of non-canonical hexamers [97]. By exploring our transcript variability, we can probably assume the absence of preferable loci for the transcription of these pericentromeric sequences. In any case, accumulation of HSat1A transcripts presumably depends on the joined complexity of transcriptional and post-transcriptional pathways [98]. In the future, the association of HSat1A with the plethora of functional significant satncRNAs would benefit from coupling our analysis with 5′ RACE or sequencing of Pol II transcription start sites (TSSs), in order to obtain deeper information regarding transcript full size and splicing complexity [99, 100].

Irrespective of being a consequence of chromosomal rearrangements and/or changes in (epi)genetic cell states, the abnormal transcription of α satellite and classical satellite sequences is a trait of tumor cells [33, 42, 50]. The present work highlights the former statement, trailing the way for the characterization of HSat1A transcripts. More functional work is crucial, possibly initiating by HSat1A knockdown and posterior evaluation of cellular phenotypes [101]: determining if this satellite is, for example, possibly involved in organizing genome architecture (as several tandem repeats) [56, 102], or in gene expression regulation during stress, development, and pathology [103]. Studying the upstream regulation of HSat1A transcripts is also essential for the mechanistic understanding of involved cellular pathways.

## Conclusions

Throughout the present study, we molecularly addressed HSat1A in multiple facades: mapping in human chromosomes, checking its organization and nuclear localization, and learning its transcription pattern and variability in different tumoral cell lines. This is the first step to functionally study HSat1A in several biological contexts, like human disease, in which pericentromeric satellite sequences seem to be frequently involved. This paper closes a lacuna in HSat transcription studies, since it proves that, like the other classical satellites (HSat2/3), HSat1A is indeed transcribed and most likely intensely deregulated depending on the gambling of cancer (epi)genetic cause-effect scenarios.

## Material and methods

### Cell culture, chromosome harvesting, genomic DNA/RNA isolation

Several human cell lines were used during this work: MCF10A, MCF7, MDA-MB-231, MDA-MB-468, SK-BR-3, A549 (ATCC-CCL-185), H1299 (ATCC-CRL-5803), PC9, H1975 (ATCC-CRL-5908), H2228 (ATCC-CRL-5935), HeLa, Caco-2, HepG2, GM12878 (Coriell Institute,), GM03417 (Coriell Institute), GM11535 (Coriell Institute), GM08854 (Coriell Institute), and HDFn (Additional file 1: Supplementary Table S6). Most cell lines were maintained in Dulbecco’s modified Eagle’s medium (DMEM)/Roswell Park Memorial Institute (RPMI) medium supplemented with 10% FBS (fetal bovine serum), 200 mM l-glutamine, and 100 µg/mL/200 µg/µL penicillin–streptomycin/neomycin antibiotic mixture. GM03417 cells were additionally supplemented with 13% AmnioMax C-100 Basal Medium and 2% AmnioMax C-100 supplement. MCF10A cells were grown in DMEM: F12 medium supplemented with 5% horse serum, 20 ng/mL epidermal growth factor (EGF), 0.5 mg/mL hydrocortisone, 100 ng/mL cholera toxin, 10 µg/mL insulin, and 100 µg/mL/200 µg/µL penicillin–streptomycin/neomycin antibiotic mixture. All the reagents mentioned above are commercialized by Gibco, Thermo Fisher Scientific. Culture conditions were maintained at 37 °C and 5% CO2, except for GM11535 (34 °C, 5% CO2) and GM08854 (37 °C, 8% CO2) cell lines. Chromosome harvesting procedures were routinely followed. Genomic DNA extraction was achieved with QuickGene DNA Tissue Kit S (Fujifilm Life Science) (instructions accordingly). RNA was isolated following the mirVana Isolation Kit (Ambion, Thermo Fisher Scientific). Total RNA was purified from DNA using the TURBO DNA-free ™ Kit (Ambion, Thermo Fisher Scientific). DNA and RNA were quantified with Qubit™ dsDNA BR Assay Kit and Qubit™ RNA BR Assay Kit, respectively. Total RNA pools from the human brain (cat. no. 636530, Takara Bio USA, Inc), small intestine (cat. no. 636539, Takara Bio USA, Inc), and mammary gland (cat. no. 636576, Takara Bio USA, Inc) were also used for the assessment of HSat1A expression in different tissues.

### HSat1A isolation, cloning, and in silico analysis

HSat1A was amplified from human genomic DNA with two sets of specific designed primers. Primers were designed using Primer 3 [104], available in Geneious R9 version 9.1.8 (Biomatters), and are described in Additional file 1: Supplementary Table S1. PCR program was as follows: initial denaturing step at 94 °C for 10 min; 30 cycles of 94 °C for 1 min (denaturation), 57 °C for 45 s (annealing), and 72 °C for 45 s (extension); final extension at 72 °C for 10 min. PCR products were run in an agarose gel and the obtained bands were isolated and cloned. Isolated HSat1A bands were purified using the QIAquick PCR Purification Kit (Qiagen). HSat1A PCR amplicons were then cloned using the vector pUC19DNA/SmaI, which requires the use of the Fast DNA End Repair (Thermo Fisher Scientific) to blunt and phosphorylate sequence ends for ligation to occur (sequences are ligated to SmaI site on pUC19 with T4 DNA ligase). Transformation was performed with DH5α competent bacterial cells (Invitrogen, Thermo Fisher Scientific). Colonies were selected with blue-white screening (β-galactosidase blue-white α complementation) and positives were confirmed by PCR. Positive clones were sequenced by Sanger methodology (STAB VIDA). Multiple sequence alignments with HSat1A clones were obtained with CLUSTAL W matrix Geneious R9 version 9.1.8 (Biomatters) [105] with default settings. Chromosome sequences from GRCh38.p14 (GenBank assembly accession GCA_000001405.29) [106] and CHM13-T2T v2.0 (GenBank assembly accession GCA_009914755.4) [107] human assemblies were downloaded and used as custom BLAST databases in Geneious R9.1.8 (Biomatters). BLAST hits were annotated in chromosomes from both assemblies. Sequence alignment between HSat1A representative clone and analyzed sequences was obtained with Geneious matrix from Geneious R9 version 9.1.8 (Biomatters) [105]; parameters were set to default values. BLAST searches from custom or NCBI databases were equally performed, setting max_target_seqs to 15,000. BLAST hits were filtrated according to the following parameters: % identity ≥ 70, *E*-value ≥ 10^−16^, query coverage ≥ 70%, and bit score ≥ 90.

For the quantification and periodicity study of HSat1A, sequencing data was extracted from the Whole Human Genome Sequencing Project, NA12878 (https://github.com/nanopore-wgs-consortium/NA12878). The header of each read was renamed by numerical order (from 1 to 15,666,888). The HSat1A repetitive sequence (SAR, accession DF0001062.4) was extracted from the Dfam database (https://dfam.org/). HSat1A clone repetitive sequence was assembled in-house. Quantification was performed using RepeatMasker [60] to detect the presence of HSAT1 (SAR) and of HSAT1 clone on reads from NA12878 sequencing data. Genomic abundance for each of the repetitive sequences was estimated based on the number of masked bases/total bases from reads × 100. For the periodicity studies of HSat1A repetitive sequences, reads with a number higher than 400 kb containing only these sequences were selected. Then, the NTRprism (https://github.com/altemose/NTRprism) scripts were used on the selected reads.

LNCipedia annotated lncRNAs from Version 5.2 Full Database [68] were downloaded (https://lncipedia.org/) and used as a custom BLAST database in Geneious.

### Metaphase fluorescent in situ hybridization (metaphase-FISH)

In order to physically map HSat1A, FISH was performed as described in [108] with slight modifications. Metaphase slides were treated with acetone for 10 min, baked at 65 °C for 1 h, and denatured in an alkaline denaturation solution (0.5 M NaOH, 1.5 M NaCl) for 1–4 min. Clone probes were PCR labeled with biotin-16-dUTP (from Roche Applied Science). Hybridization was performed overnight. Post-hybridization stringency was done with 1 × SSC at 73 °C. Biotin-labeled HSat1A probe was detected with streptavidin Cy3 conjugated (Sigma-Aldrich). Preparations were mounted using Vectashield containing 4′-6-diamidino-2-phenylindole (DAPI) (Vector Laboratories) to counterstain chromosomes.

FISH with human acrocentric chromosome paint probes was performed sequentially to the hybridization with the HSat1A probe. Briefly, FISH slides were washed in 2 × SSC for 10 min, followed by denaturation in an alkaline denaturation solution (0.5 M NaOH, 1.5 M NaCl) for 2–5 min. The paint probes were labeled by DOP-PCR as follows: human 13 was labeled with Atto-488-dUTPs, human 14 with Atto425-dUTPs, human 21 with Atto-Cy5xx-dUTPs, human 22 with Atto-594-dUTPs, and human 15 with Atto-425-dUTPs and Atto-594-dUTPs (Jena Bioscience). Hybridization was carried out overnight at 37 °C. Post-hybridization washes were done as described above.

FISH images were observed using a Zeiss ImagerZ2 microscope coupled to an ORCA-Flash 4.0 digital camera (Hamamatsu) and captured using SmartCapture 4 software (Digital Scientific, UK). Digitized photos were prepared for printing in Adobe Photoshop (version 7.0).

### RNA-FISH/RNA-FISH-IF/sequential DNA-FISH.

RNA-FISH was performed according to [64], with some modifications. Cells were hybridized overnight at 37 °C with the PCR-Biotin-labeled HSat1A probe. Probe detection was carried out with streptavidin, Cy3 conjugated, or using Invitrogen™ Alexa Fluor™ 555 Tyramide SuperBoost™ Kit, streptavidin (Thermo Fisher Scientific), according to provided instructions. The second approach was used in cell lines with smaller amounts of HSat1A transcripts (not visible with standard detection methods). When RNA-FISH experiments were coupled with immunofluorescence (IF) with anti-fibrillarin antibody, the RNA-FISH protocol was performed as above (with paraformaldehyde fixation for preserving nuclear structure). Primary incubation was performed for 1 h (anti-fibrillarin monoclonal mouse, 1:100, MA3-16,771, Thermo Scientific), followed by incubation with secondary antibody (anti-mouse monoclonal FITC, 1:200, 81–6511, Zymed). Cells were then mounted with coverslips and counterstained with Vectashiled mounting medium containing 4′-6-diamidino-2-phenylindole (DAPI) (Vector Laboratories). RNase A treatments (0.8 mg/mL, Sigma-Aldrich) were performed after permeabilization for 3 h at 37 °C. Analysis of the average intensity of active objects was performed in 20 cells from control RNA hybridization and RNase treatment in AutoQuant X3 software (Media Cybernetics), “Counting and Tracking” tool in all slices. For the sequential DNA-FISH, RNA-FISH slides were washed in 2 × SSC for 10 min, treated with RNAse A (100 μg/mL in 2 × SSC) for 30 min at 37 °C, dehydrated through an ethanol series, denatured in 70% formamide/2 × SSC for 2 min at 72 °C, and hybridized overnight with HSat1A probe labeled with Atto488-dUTPs (Jena Bioscience). Post-hybridization stringent washes were done with 0.1 × SSC at 42 °C. Slides were mounted with a Vectashiled mounting medium containing 4′-6-diamidino-2-phenylindole (DAPI) (Vector Laboratories).

For RNA-FISH/RNA-FISH-IF/sequential DNA-FISH images, confocal fluorescence imaging was performed on an LSM 510 META with a Zeiss Axio Imager Z1 microscope and LSM 510 software (version 4.0 SP2). Applied settings were constant. Used lasers were argon (488 nm) set at 12.9%, helium–neon (543 nm) set at 50.8%, and diode (405 nm) set at 9.9%. The pinhole was set to 96 mm (1.02 airy units) for the argon laser, 102 mm (0.98 airy units) for the helium–neon laser, and 112 mm for the diode laser using a 63 × objective. Images were captured at a scan speed of 5 with 1-μm-thick Z sections. AutoQuant X3 software (Media Cybernetics) allowed 3D deconvolution. Subsequent TIFF processing was run in ImageJ (1.52v). The three-dimensional isosurfaces and orthogonal slices (perpendicular or parallel angles) were produced in Image Pro Premier 3D (version 10, Media Cybernetics).

### DNA copy number quantification (quantitative polymerase chain reaction (qPCR))/RNA expression analysis by real-time reverse transcriptase–qPCR (RT-qPCR)

HSat1A copy number evaluation was performed using a standard curve obtained with serial dilutions of the recombinant plasmid (with HSat1A clone). This method allowed to interpolate C_T_ values (obtained in different DNA samples) against the standard curve. Real-time qPCR reactions were performed with MeltDoctor HRM Master Mix (Applied Biosystems, Thermo Fisher Scientific), according to the manufacturer’s protocol. The standard curve method was also used for HSat1A ncRNA quantification, as previously validated [109]. Quantification was carried out using SensiFAST™ SYBR® Hi-ROX One-Step Kit (Bioline). RNA quantification was obtained by interpolating its CT value against the standard curve. All reactions used 100 ng of RNA and all data were normalized to MCF10A.

Primers, standard curve parameters, and PCR programs for both experiments are present in Additional file 1: Supplementary Table S7. Primer specificity was always evaluated by the generation of a melt curve. All reactions were performed in triplicate, and negative controls were also included in the plate. Data were analyzed using the same parameters on the StepOne software (version 2.2.2, Applied Biosystems, Thermo Fisher Scientific). All data are presented as mean ± standard deviation. Statistical significance was determined using ANOVA tests: ns (non-statistically significant) for *p* > 0.05, *p ≤ 0.05, **p ≤ 0.01, ***p ≤ 0.001, ****p ≤ 0.0001.

### 3′ RACE, RACE-Seq, and sequencing analysis

For further characterization of HSat1A transcripts (size and poly-A tail), the 5′/3′ RACE Kit, 2nd Generation (Roche), was followed. HeLa cDNA was prepared using the kit and subjected to PCR using the provided PCR anchor primer and the HSat1A forward primer. 3′ RACE was coupled with high-throughput sequencing, performed by STAB VIDA NGS sequencing service. The analysis of the generated sequence raw data was carried out using CLC Genomics Workbench 12.0.3 (https://www.qiagenbioinformatics.com/). The following data processing is detailed in Additional file 1: Supplementary Fig. S6. RACE-PCR was followed by paired-end 300-nt Illumina sequencing. To evaluate the quality of the reads throughout the workflow, FASTQC [110] was used. CutAdapt [111] was used to remove the Universal Adaptors on the extremities of the reads. The paired reads were merged using the PEAR [112] tool. To guarantee the quality of the reads, a score Phred of equal or higher than 30 was applied with the tool Seqkit [113]. Furthermore, to also guarantee that at least one monomer of HSat1A was present on every read, selection for the size of the sequences to be at least 85 bp or higher was applied, considering the downstream removal of the Oligo Anchor primer of the reads. For the next step, all the reads were set in the same orientation, to ensure that we could proceed with a Multiple Sequence Alignment analysis downstream. This was also done with the tool Seqkit [113] and the GNU grep (https://www.gnu.org/software/grep/manual/grep.html). Then, the identification of reads containing the HSat1A (SAR) satellite sequence was performed with RepeatMasker [60]. The count and removal of duplicates within these reads containing the HSat1A (SAR) satellite sequence were performed using the Seqkit [113] tool. Subsequently, clustering of these sequences was achieved with the MESHClust v3.0 program [114], selecting for a threshold identity score of 90% to determine the cluster membership. Multiple sequence alignment was performed with a Rscript [115] on cluster center sequences to produce a dendrogram. From the clustering, it was possible to discover motifs based on the sequence in each group using the Improbizer tool (http://hgdownload.soe.ucsc.edu/admin/exe/linux.x86_64/ameme and https://users.soe.ucsc.edu/~kent/improbizer/improbizer.html). We restricted the identification of motifs to a maximum of 6 motifs the tool could find, with a least one motif with same length or higher than 7 base pairs, and for a maximum of occurrences of the motifs within each sequence for 1 and 3. For representation of the sequences, the probability of every nucleotide in each position was considered. Applying these metrics with a R package, ggseqlogo [116], was possible to identify which nucleotide was more prevalent in each position, for each sequence selected (not shown). A merged alignment of the HSat1A monomer sequence and the detected motif sequences in each cluster group was performed with the NCBI Genome Workbench [117] tool. The scripts and produced data are publicly available at https://github.com/GamaPintoLab/HSAT1A-transcript-analysis.

## Supplementary Information

**Additional file 1: Supplementary Table S1.** Primer sequences for HSat1A isolation. The first set of primers was also used for qPCR and RT-qPCR reactions. **Supplementary Table S2.** Quantification of repetitive sequences HSat1A and HSat1A_clone6 based on NA12878 sequencing data. *Genomic abundance is an estimation based on the total number of bp on all the reads, 15,666,888 bp. **Supplementary Table S3.** Distance matrix (% identity) of the alignment between representative HSat1A clones, L01057.1 (extracted region), and CP068265.2 (extracted region). Distances were calculated using Geneious alignment (version 9.1.8, Biomatters). **Supplementary Table S4. **Analysis of the average intensity of active signal objects (all slices) in RNA-FISH control and RNA-FISH with RNase A treatment, performed in ‘Counting and Tracking, AutoQuant X3 (Media Cybernetics). Data presented as mean ± SD for the analysis of 20 cells. **Supplementary Table S5.** Sequencing run statistics for 3’RACE-Seq (Whole Genome Library Preparation, Illumina MiSeq plataform, NGS Sequencing service STAB VIDA). **Supplementary Table S6.** Cell lines description table, specifying tissue and type (tumoral and non-tumoral). **Supplementary Table S7.** Standard curves parameters and PCR programs for both reactions (DNA copy number qPCR and RNA RT-qPCR). **Supplementary Figure S1.** Visual representation of the obtained BLAST hits from Supplementary Table S3 (query: HSat1A clone; Database: nt). A total of 8167 hits is distributed for 47 sequences, 38 of which (5000 hits) belong to unlocalized sequences from a sequencing project for the “Construction and Integration of Three De Novo Japanese Human Genome Assemblies toward a Population-Specific Reference” (BioSample: SAMD00243993; Bioproject: PRJDB10452) [119, 120]. CP068257.2, CP068256.2, and CP068263.2 represent accessions from CHM13 T2T v2.0 (GCA_009914755.4) human assembly. **Supplementary Figure S2.** Statistical analysis (one-way ANOVA with Tukey ‘s multiple comparisons test) of monomer copy number (data from supplementary Table S6). *P≤0.05, **P≤0.01, ***P≤0.001, ****P≤0.0001, ns - not statistically significant. **Supplementary Figure S3.** HSat1A FISH mapping (red) in chromosome 1 (blue). Signal hybridization in chromosome 1 is visible in GM12878, MCF10A, HDFn, and GM03417 (non-tumoral cell lines). **Supplementary Figure S4.** Original uncropped gel for Figure 4A; the box indicates where the gel was cropped. **Supplementary Figure S5.** Comparison between the HSat1A monomer sequence and a selection of motifs sequences in each cluster group through a merged alignment. **Supplementary Figure S6.** Workflow for the analysis of HSat1A RACE-Seq.

**Additional file 2.** In silico data of HSat1A BLAST searches. Obtained hits by chromosome from GRCh38.p14 (GCA_000001405.29) and CHM13 T2T v2.0 (GCA_009914755.4) human assemblies.

**Additional file 3.** HSat1A clone BLAST against NCBI nucleotide database (default parameters, except maximum number of hits). Results are presented by accession numbers and best matching hit for each accession.

**Additional file 4.** Copy number of HSat1A monomer by cell line presented in monomer copy; Relative quantification of HSat1A transcripts in the analyzed cell lines. MCF10A was considered the reference.

**Additional file 5.** BLAST search of HSat1A 3’ RACE-seq on-target mapped reads against LNCipedia 5.2 Full Database and SRA NCBI SRX250554 (in silico data for filtrated BLAST hits)

**Additional file 6.** Overlap analysis of HSat1A transcript reads, according to read length and abundance. The largest most abundant read is highlighted and served as representative of poly(A) site analysis (displayed in main text Fig. 4C).

## Data Availability

All data generated or analyzed during this study are included in this published article, its supplementary information files, and publicly available repositories. Supporting data values are available in additional files. Sequence data for HSat1A clones is available in GenBank under accession numbers: OP172545–OP172627. The 3′ RACE-Seq data generated in this study have been submitted to the NCBI BioProject database (https://www.ncbi.nlm.nih.gov/bioproject/) under accession number PRJNA867346 [118].
