# Supplementary material for: Human Satellite 1A analysis provides evidence of pericentromeric transcription
Source: BMC Biol. 2023 Feb 8;21:28. doi: 10.1186/s12915-023-01521-5 (PMC9909926; doi:10.1186/s12915-023-01521-5)
Supplement: Supplementary file 1 — Additional file 1: Supplementary Table S1. Primer sequences for HSat1A isolation. The first set of primers was also used for qPCR and RT-qPCR reactions. Supplementary Table S2. Quantification of repetitive sequences HSat1A and HSat1A_clone6 based on NA12878 sequencing data. *Genomic abundance is an estimation based on the total number of bp on all the reads, 15,666,888 bp. Supplementary Table S3. Distance matrix (% identity) of the alignment between representative HSat1A clones, L01057.1 (extracted region), and CP068265.2 (extracted region). Distances were calculated using Geneious alignment (version 9.1.8, Biomatters). Supplementary Table S4. Analysis of the average intensity of active signal objects (all slices) in RNA-FISH control and RNA-FISH with RNase A treatment, performed in ‘Counting and Tracking, AutoQuant X3 (Media Cybernetics). Data presented as mean ± SD for the analysis of 20 cells. Supplementary Table S5. Sequencing run statistics for 3’RACE-Seq (Whole Genome Library Preparation, Illumina MiSeq plataform, NGS Sequencing service STAB VIDA). Supplementary Table S6. Cell lines description table, specifying tissue and type (tumoral and non-tumoral). Supplementary Table S7. Standard curves parameters and PCR programs for both reactions (DNA copy number qPCR and RNA RT-qPCR). Supplementary Figure S1. Visual representation of the obtained BLAST hits from Supplementary Table S3 (query: HSat1A clone; Database: nt). A total of 8167 hits is distributed for 47 sequences, 38 of which (5000 hits) belong to unlocalized sequences from a sequencing project for the “Construction and Integration of Three De Novo Japanese Human Genome Assemblies toward a Population-Specific Reference” (BioSample: SAMD00243993; Bioproject: PRJDB10452) [119, 120]. CP068257.2, CP068256.2, and CP068263.2 represent accessions from CHM13 T2T v2.0 (GCA_009914755.4) human assembly. Supplementary Figure S2. Statistical analysis (one-way ANOVA with Tukey ‘s multiple comparisons test) of monomer co [file 12915_2023_1521_MOESM1_ESM.docx]

**Additional File 1**

**Supplementary Table S1.** Primer sequences for HSat1A isolation. The first set of primers was also used for qPCR and RT-qPCR reactions.

|  |  | Accession Number Primer Design |
| --- | --- | --- |
| HSat1A F1 | 5’-TGTGCGGTACATAAGATATCAAAG-3’ | JX174276.1 |
| HSat1A R1 | 5'-AAGTACCCAACCATATTT-3' |  |
| HSat1A F2 | 5’-TGTGCAGCATGTAATATGAA-3’ | L01057.1 |
| HSat1A R2 | 5’-ACGTTGCATAAACTATCAAA-3’ |  |

**Supplementary Table S2.** Quantification of repetitive sequences HSat1A and HSat1A_clone6 based on NA12878 sequencing data. *Genomic abundance is an estimation based on the total number of bp on all the reads, 15,666,888 bp.

| Satellite Family | Monomer (bp) | Masked (bp) | Element Count | Genomic Abundance* (%) |
| --- | --- | --- | --- | --- |
| HSat1A (SAR) | 84 | 284,538,738 | 1,376,831 | 0.2140 |
| HSat1A Clone | 210 | 337,528,371 | 175,971 | 0.2539 |

**Supplementary Table S3.** Distance matrix (% identity) of the alignment between representative HSat1A clones, L01057.1 (extracted region), and CP068265.2 (extracted region). Distances were calculated using Geneious alignment (version 9.1.8, Biomatters).

|  | L01057.1 | HSat1A Clone (1) | HSat1A Clone (2) | HSat1A Clone (3) |
| --- | --- | --- | --- | --- |
| CP068265.2 | 77.3 | 80.5 | 80.1 | 79.1 |
| L01057.1 |  | 93.8 | 81.8 | 82.9 |
| HSat1A clone (1) |  |  | 82.3 | 82.9 |
| HSat1A clone (2) |  |  |  | 95.7 |

**Supplementary Table S4.** Analysis of the average intensity of active signal objects (all slices) in RNA-FISH control and RNA-FISH with RNase A treatment, performed in ‘Counting and Tracking, AutoQuant X3 (Media Cybernetics). Data presented as mean ± SD for the analysis of 20 cells.

|  | Average Intensity (Active Objects) |
| --- | --- |
| RNA-FISH HSat1A_H1299_control | 2004 (±265.1) |
| RNA-FISH HSat1A_H1299_RNase A | 464.1 (±67.20) |

**Supplementary Table S5.** Sequencing run statistics for 3’RACE-Seq (Whole Genome Library Preparation, Illumina MiSeq plataform, NGS Sequencing service STAB VIDA).

| Number Of Bases (Raw Data) [Mbp] | Number Of Reads (Raw Data) | Mean Read Length [Bp] |
| --- | --- | --- |
| 59.27 | 196928 | 300 |

**Supplementary Table S6.** Cell lines description table, specifying tissue and type (tumoral and non-tumoral).

| Cell Line | Tissue | Type | Details |
| --- | --- | --- | --- |
| GM12878 | Human blood | Non-tumoral | Epstein-Barr Virus (EBV) transformed lymphoblastoid line |
| GM03417 | Human fibroblasts | Non-tumoral | Mosaic; 32% of cells are balanced 45,XX,rob(14;21) |
| HDFn | Human skin | Non-tumoral |  |
| MCF10A | Human breast | Non-tumoral |  |
| HeLa | Human cervix | Adenocarcinoma |  |
| A549 | Human lung | Adenocarcinoma |  |
| H1299 | Human lung | Carcinoma |  |
| H1975 | Human lung | Adenocarcinoma |  |
| H2228 | Human lung | Adenocarcinoma |  |
| PC9 | Human lung | Adenocarcinoma |  |
| MCF-7 | Human breast | Carcinoma |  |
| MDA-MB-231 | Human breast | Adenocarcinoma |  |
| MDA-MB-468 | Human breast | Adenocarcinoma |  |
| SK-BR-3 | Human breast | Adenocarcinoma |  |
| Caco-2 | Human colon | Adenocarcinoma |  |
| Hep-G2 | Human liver | Hepatoblastoma |  |
| GM08854 | Mouse/human somatic cell hybrid | Non-tumoral | Retains human chr21 |
| GM11535 | Human/Chinese hamster somatic cell hybrid | Non-tumoral | Retains human chr14 |

**Supplementary Table S7.** Standard curves parameters and PCR programs for both reactions (DNA copy number qPCR and RNA RT-qPCR).

| DNA | R^2^ | Efficiency % | Program |
| --- | --- | --- | --- |
|  | 0.995 | 108.802 | Initial denaturation at 95ºC (10 Min); 40 cycles of 15s (95ºC) and 1 min (58ºC); Melt curve with a denaturation step of 15s (95ºC), annealing for 1 Min (58ºC); High-Resolution Melting – 95ºC (15s) with a continuous ramp rate of 0,3%. |
| RNA | 0.994 | 108.395 | Initial reverse transcriptase reaction at 45ºC (20 min); Denaturation at 95ºC (2 min); 40 cycles Of 5s (95ºC) and 20s (58ºC). |

**Supplementary Figures**


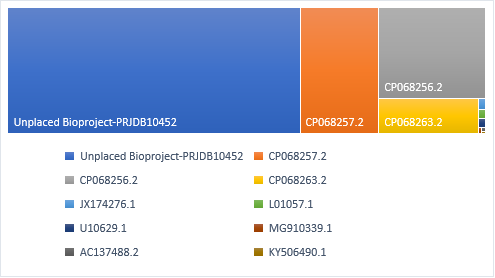


**Supplementary Figure S1.** Visual representation of the obtained BLAST hits from Supplementary Table S3 (query: HSat1A clone; Database: nt). A total of 8167 hits is distributed for 47 sequences, 38 of which (5000 hits) belong to unlocalized sequences from a sequencing project for the “Construction and Integration of Three De Novo Japanese Human Genome Assemblies toward a Population-Specific Reference” (BioSample: SAMD00243993; Bioproject: PRJDB10452). CP068257.2, CP068256.2, and CP068263.2 represent accessions from CHM13 T2T v2.0 (GCA_009914755.4) human assembly.

**Supplementary Figure S2.** Statistical analysis (one-way ANOVA with Tukey ‘s multiple comparisons test) of monomer copy number (data from supplementary Table S6). *P≤0.05, **P≤0.01, ***P≤0.001, ****P≤0.0001, ns - not statistically significant.


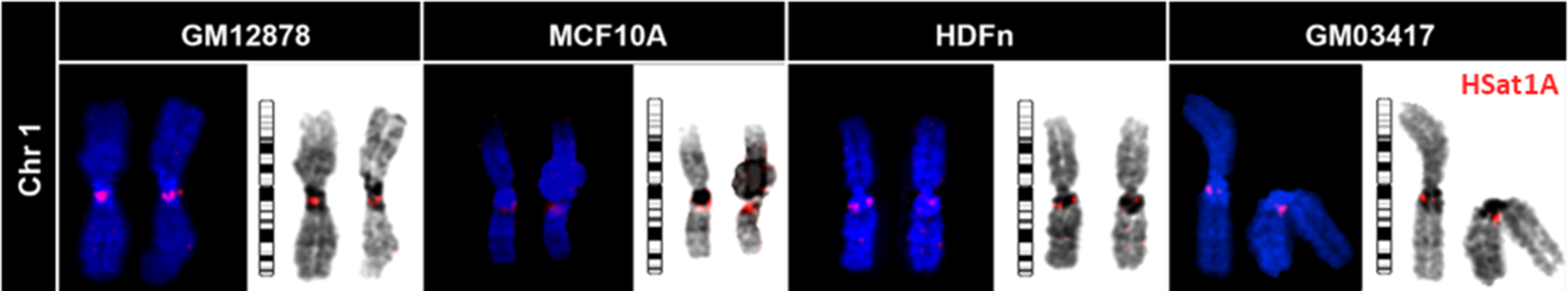


**Supplementary Figure S3.** HSat1A FISH mapping (red) in chromosome 1 (blue). Signal hybridization in chromosome 1 is visible in GM12878, MCF10A, HDFn, and GM03417 (non-tumoral cell lines).


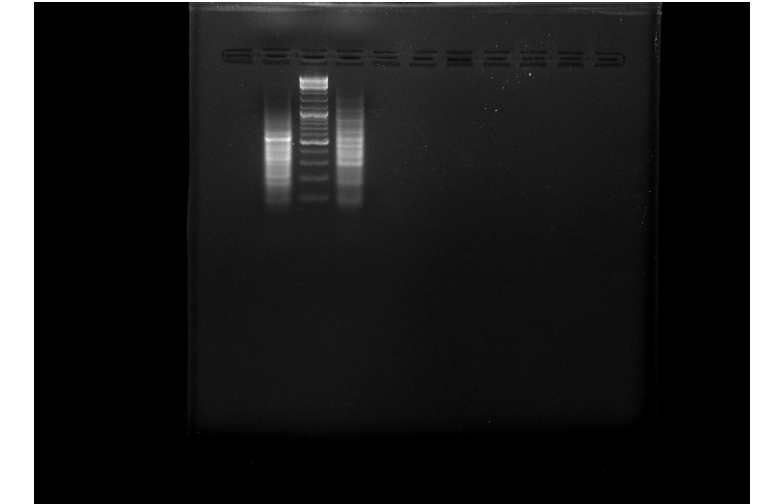


**Supplementary Figure S4.** Original uncropped gel for Figure 4A; the box indicates where the gel was cropped.


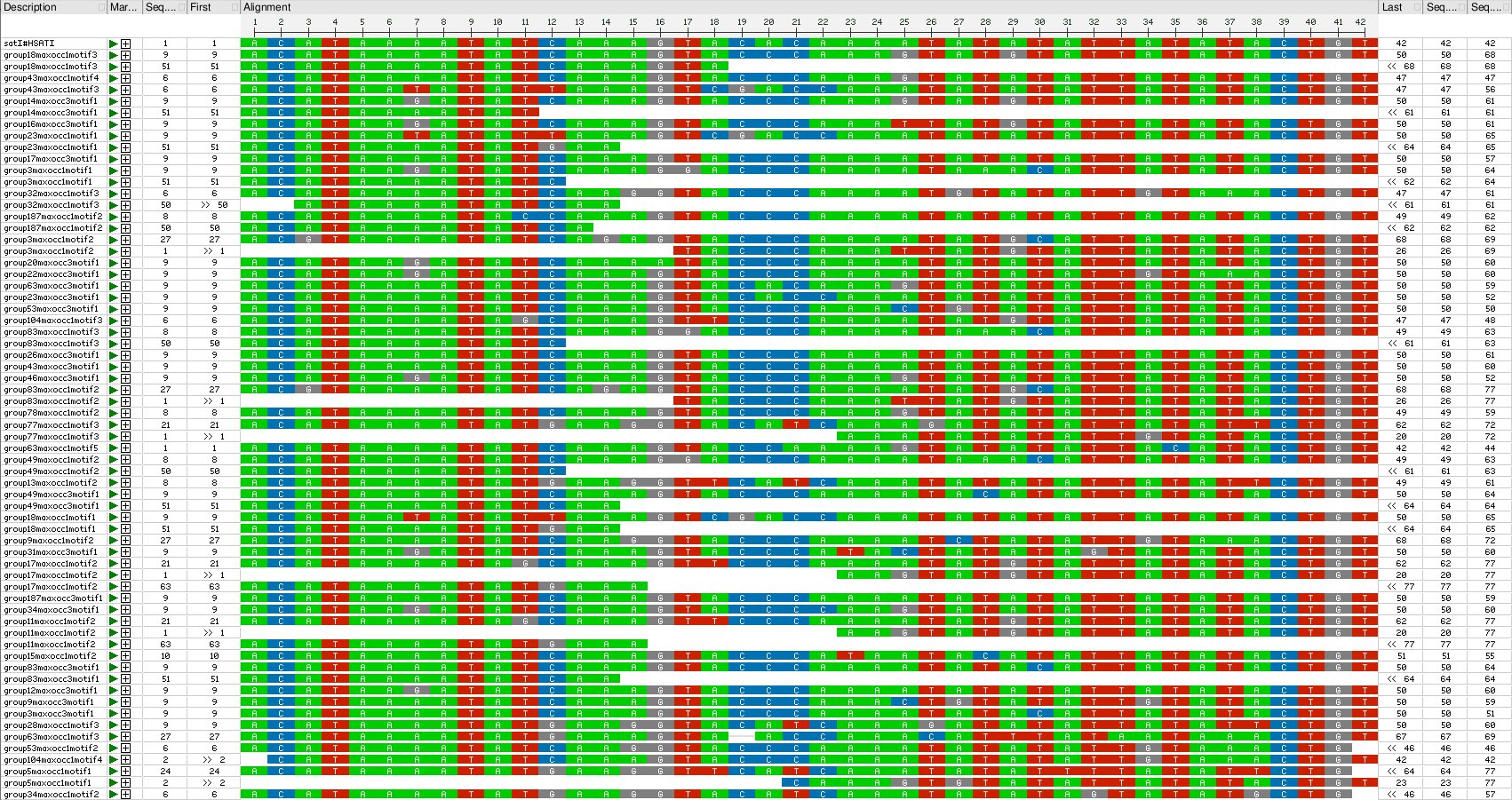


**Supplementary Figure S5.** Comparison between the HSat1A monomer sequence and a selection of motifs sequences in each cluster group through a merged alignment.


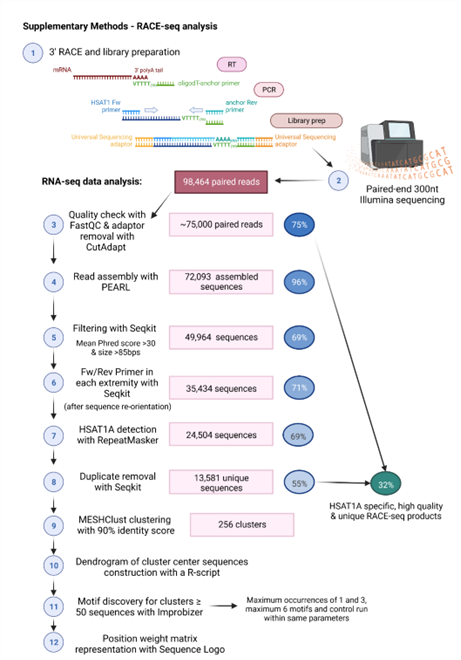


**Supplementary Figure S6.** Workflow for the analysis of HSat1A RACE-Seq.
